# Effects of Customized Digital Health Care Service on Metabolic Syndrome Status and Lifestyle Using a Health Care App: Clinical Trial

**DOI:** 10.2196/41427

**Published:** 2023-01-18

**Authors:** ChulYoung Bae, Bo-Seon Kim, KyungHee Cho, Ji-Hyun Kim, In-Hee Kim, Jeong-Hoon Kim

**Affiliations:** 1 Mediage Research Center Seongnamsi Republic of Korea; 2 Department of Family Medicine National Health Insurance Service Ilsan Hospital Goyang Republic of Korea

**Keywords:** biomarkers, health care, lifestyle, metabolic syndrome, telemedicine

## Abstract

**Background:**

Untact cultures have rapidly spread around the world as a result of the prolongation of the COVID-19 pandemic, leading to various types of research and technological developments in the fields of medicine and health care, where digital health care refers to health care services provided in a digital environment. Previous studies relating to digital health care demonstrated its effectiveness in managing chronic diseases such as hypertension and diabetes. While many studies have applied digital health care to various diseases, daily health care is needed for healthy individuals before they are diagnosed with a disease. Accordingly, research on individuals who have not been diagnosed with a disease is also necessary.

**Objective:**

This study aimed to identify the effects of using a customized digital health care service (CDHCS) on risk factors for metabolic syndrome (MS) and lifestyle improvement.

**Methods:**

The population consisted of 63 adults who underwent a health checkup at the National Health Insurance Service Ilsan (NHIS) Hospital in 2020. Measured variables include basic clinical indicators, MS-related variables, and lifestyle variables. All items were measured at NHIS Ilsan Hospital before the use of the CDHCS and 3 months thereafter. The CDHCS used in this study is a mobile app that analyzes the health condition of the user by identifying their risk factors and provides appropriate health care content. For comparison between before and after CDHCS use (pre-post comparison), paired *t* test was used for continuous variables, and a chi-square test was used for nominal variables.

**Results:**

The study population included 30 (47.6%) male and 33 (52.4%) female participants, and the mean age was 47.61 (SD 13.93) years. The changes in clinical indicators before and after intervention results showed a decrease in weight, waist circumference, triglyceride, and high-density lipoprotein cholesterol and increases in systolic blood pressure and diastolic blood pressure. The distribution of the risk group increased from 32 (50.8%) to 34 (54%) and that of the MS group decreased from 18 (28.6%) to 16 (25.4%). The mean metabolic syndrome age–chronological age before the CDHCS was 2.20 years, which decreased to 1.72 years after CDHCS, showing a decrease of 0.48 years in the mean metabolic syndrome age–chronological age after the intervention. While all lifestyle variables, except alcohol consumption, showed a tendency toward improvement, the differences were not statistically significant.

**Conclusions:**

Although there was no statistical significance in the variables under study, this pilot study will provide a foundation for more accurate verification of CDHCS in future research.

## Introduction

Metabolic syndrome (MS) refers to a cluster of diseases appearing simultaneously in an adult and is typically defined as having 3 or more of the following 5 indicators: systolic blood pressure (SBP), fasting blood sugar (FBS), triglyceride (TG), high-density lipoprotein cholesterol (HDL-C), and waist circumference (WC) belonging to the abnormal range [1]. The causes of MS include inappropriate dietary habits, lack of physical activity, and alcohol consumption [2-5]. According to a 2005 survey among Korean adults, the prevalence of MS was 20.3%, indicating that 1 in 5 Korean adults had MS. As MS is mostly associated with lifestyle factors, it can be prevented through lifestyle improvement. There is a growing need for such preventive methods and real-life health care associated with MS [6-9].

*Untact* culture has spread rapidly around the world as a result of the COVID-19 pandemic. In particular, various types of research and technological developments are taking place in the medical and health care fields in light of related social issues. There is active research in various fields of digital health care, including digital therapeutics (DTx), and information and communication technologies, while technologies are also evolving accordingly [10,11].

DTx is defined as a scientific evidence-based treatment method operated by high-quality software technologies for preventing, managing, and treating disorders or diseases [12]. It is a new concept of health care service that is rapidly emerging as a form of new treatment for the prevention, management, and treatment of chronic disease and behavioral modification [13-15]. DTx tools include multiple screen devices such as smartphones, tablets, computers, and video game platforms that converge with software algorithms and can be applied to the improvement of treatment management and rehabilitation [16]. This non–face-to-face health care technology is a time- and cost-effective way for both medical staff and individuals because users do not need to visit the hospital every time [17]. Therefore, the continuous development and development of the field can be seen as an important social value, and the key reason why such digital health care is attracting attention is that its goal is to be “predictive, preventive, personalized, and participatory” (P4) [18].

Previous studies relating to digital health care demonstrated its effectiveness in managing chronic diseases such as hypertension, diabetes, asthma, cystic fibrosis, and cessation of smoking [19-25]. Previous studies also reported the effectiveness of digital health care for various syndromes and diseases [26-29]. While many studies have applied digital health care to various diseases, daily health care is needed for healthy individuals before they are diagnosed with a disease. Accordingly, research on individuals who have not been diagnosed with a disease is also necessary. Therefore, we believe that a basic experimental study, such as this study, can be the basis for further research.

MS does not refer to a single disease but a cluster of symptoms; therefore, there is low awareness about it. Most people who meet the criteria for MS do not recognize this fact. Therefore, it is important to evaluate the severity of MS and to present the results in an easy-to-understand manner. In many previous studies on digital health care, health care coaching has been conducted using mobile messaging services (SMS text messaging) or telephone calls, whereas this study aimed to identify the effects of digital health care using a customized digital health care service (CDHCS), which provides a service tailored to the user’s needs after considering their physical and physiological characteristics. The app synthesizes the collected data to analyze the MS age (MSA), while also providing content regarding physical activities, and nutritional, mental, and medical knowledge.

We aimed to study the effects of using a CDHCS for 3 months on MS risk factors and lifestyle improvement. This study was conducted as a pilot study for verification of the effectiveness of the CDHCS prior to a main study to be conducted in the future.

## Methods

### Study Design and Participants

**Figure 1:**
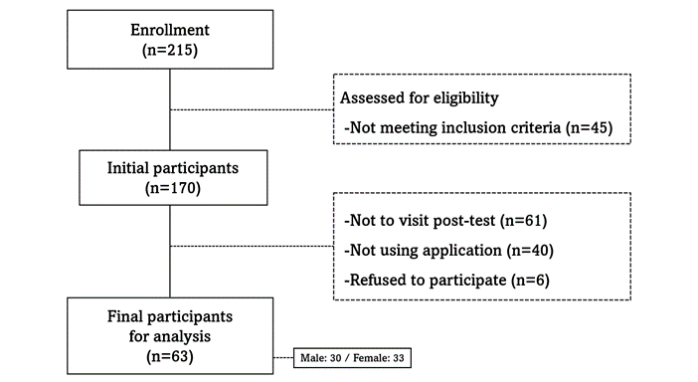
Flowchart demonstrating the participant selection process.

This clinical trial followed a one-group pre- and posttest design. The study period was June-October 2020, during which a 3-month intervention was applied to all participants. The initial study population included 215 individuals who volunteered to participate and who had taken part in the 2020 National Health Insurance Service (NHIS) health examination. The inclusion criteria were as follows: Korean adults aged ≥20 years, individuals who received NHIS health examinations at Ilsan Hospital in 2020, individuals able to use smart devices, and individuals without cancer or severe rare diseases. After the preliminary eligibility assessment, 45 individuals were excluded. Then, the following individuals were excluded from the final study population: 61 individuals who did not attend the postintervention evaluation (posttest), 40 individuals who did not access the application during the intervention period, and 6 individuals who withdrew their consent to participate. Ultimately, the final analysis was performed using data from 63 participants (30 males and 33 females; Figure 1).

### Ethics Approval

This study was approved by the institutional review board of NHIS Ilsan Hospital (NHIMC 2020-02-002). All participants received verbal explanations regarding the study and submitted their prior written consent. The study was conducted using only data without personal identification information. Participants in the study were not rewarded for their participation.

### Outcome Measurements

All items were measured at NHIS Ilsan Hospital before the use of the CDHCS and 3 months thereafter. The step count variable was measured in real time by a mobile phone app, and the analysis was performed using the average of the values from the first and last month.

### Clinical Indicators

The variables collected for data analysis were as follows: height, weight, WC, SBP, and diastolic blood pressure (DBP). Blood analysis was performed using samples collected in the morning after at least 8 hours of fasting. The analyzed markers were FBS, TG, and HDL-C [30].

### Variables Related to MS

The groups were divided by evaluating how many of the following 5 criteria were applied: (1) obesity (WC): more than 90 cm for males and more than 85 cm for females; (2) low HDL-C: less than 40 mg/dL for males and less than 50 mg/dL for females or taking medication for this condition; (3) hypertriglyceridemia (TG): more than 150 mg/dL or taking medication for this condition; (4) hypertension (SBP/DBP): more than 130/85 mm/Hg or taking medication for this condition; and (5) hyperglycemia (FBS): more than 100 mg/dL or taking medication for this condition. Individuals who meet 3 or more of the above 5 criteria were assigned to the MS group. Individuals who met 1 or 2 were placed in the risk group, and individuals who met none of the criteria were placed in the normal group [30]. We grouped the participants to determine whether the factors related to MS would change and confirmed the distribution of these groups.

### MSA and Classification

MSA refers to the age calculated through biomarkers related to MS and indicates the severity of MS. The application used the following equation to automatically calculate MSA based on the biomarkers of the user and the results were displayed on the screen [31].


Male = – 82.688 + 0.779x (WC) + 0.227x (pulse pressure) + 0.269x (FBS) + 0.085x (TG) – 0.481x (HDL-C) + 0.857x (chronological age) **(1)**

Female = – 60.340 + 0.613x (WC) + 0.371x (pulse pressure) + 0.328x (FBS) + 0.100x (TG) – 0.385x (HDL-C) +0.538x (chronological age) **(2)**

This was used to classify each type of MSA to provide differentiated feedback based on the severity of the MS. In addition, the stage was classified using the difference value between the chronological age (CA) and the metabolic syndrome age–chronological age (MSA-CA): more than +1=“good,” between 1 and +1=“average,” less than 1=“poor.”

### Lifestyle Variables

The following lifestyle variables were measured using a questionnaire survey: frequency of exercise, smoking history, weekly alcohol consumption, sleep duration, stress levels, and dietary habits. Smoking history was investigated by classifying the participants as nonsmokers and current smokers. Alcohol consumption was investigated based on the number of drinks per week and the amount of alcohol consumed per day (glasses) among participants who were identified as drinkers, and the findings were used to calculate the weekly alcohol consumption using the equation “number of drinks in a week (frequency) × amount of alcohol (ml) × alcohol content × 0.785 (specific gravity of alcohol)/100” (Suk, unpublished data, 2018). Sleep duration was categorized into 3 groups: <7, 7-8, and >8 hours of sleep. For stress levels, subjective stress levels were graded on a 5-point Likert scale (1: very low stress; 2: low stress; 3: moderate stress; 4: high stress; 5: very high stress). Dietary habits were arbitrarily categorized as good or bad based on habits related to obesity, hypertension, hyperlipidemia, and diabetes.

### Intervention Method: CDHCS

**Figure 2:**
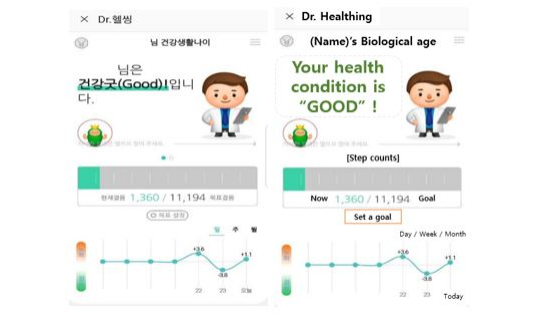
Customized digital health care service app screen (example view).

The CDHCS used in this study, called Dr Healthing, was developed by the Mediage Research Institute. Dr Healthing provides information regarding the health status of individuals by analyzing the data linked to health checkups from medical institutions and average daily step count data from a Samsung Health app. With respect to the mobile health care contents used, health checkup results from the same sex and age groups were compared and the poorest factors were selected. Then, subjects were assigned to 5 management groups: obesity, blood pressure, blood sugar, lipid, and normal group. Participants were assigned to these groups based on the poorest value of the clinical indicator, meaning participants assigned to the obesity group had WC as their poorest indicator, those in the blood pressure group had the poorest SBP or DBP, those in the blood sugar group has the poorest FBS, and those in the lipid group had the poorest TG or HDL-C. Individuals with no risk factors were assigned to the normal group. According to the customized groups assigned in this manner, health coaching contents were provided 3 times a week: day 1 (Tuesday 2 PM)—nutrition/exercise/mental coaching; day 2 (Thursday 2 PM)—general medical knowledge; and day 3 (Sunday 9 AM)—individualized weekly health report. It was possible to set the target number of steps according to the MSA, and by providing weekly/monthly summary reports, it was possible to provide feedback on health care/nutrition components necessary for each individual on a daily basis, while also being able to check on their own the changes in MSA (Figure 2).

### Statistical Analysis

Participants’ characteristics were presented as mean (SD) for continuous variables and frequency and percentage for nominal variables. For comparison between before and after CDHCS use (pre-post comparison), paired *t* test was used for continuous variables, and a chi-square test was used for nominal variables. The analysis was performed using Python version 3.8.3 software (Python Software Foundation) with statistical significance set to 0.05.

## Results

The study population included 30 (47.6%) male and 33 (52.4%) female participants, and the mean age was 47.61 (13.93) years.

### Changes in Clinical Indicators Before and After Intervention

Changes in clinical indicators before and after 3 months were presented as mean (SD). The results showed a decrease in weight, WC, TG, and HDL-C and increases in SBP and DBP after the intervention. All indicators tended to show improvement, although the differences were not statistically significant (Table 1).

**Table 1:** Changes in clinical indicators before and after CDHCS use.

Parameters	Before CDHCS^a^, mean (SD)	After CDHCS, mean (SD)	*P* value
Height (cm)	164.80 (9.07)	164.76 (9.03)	.34
Weight (kg)	66.86 (13.36)	66.72 (13.08)	.52
Waist circumference (cm)	79.65 (10.08)	79.34 (9.03)	.60
Systolic blood pressure (mmHg)	123.74 (11.53)	124.79 (10.22)	.44
Diastolic blood pressure (mmHg)	75.39 (9.18)	75.49 (7.80)	.94
Fasting blood sugar (mg/dL)	99.22 (13.20)	97.52 (11.20)	.15
Triglycerides (mg/dL)	129.68 (101.69)	109.19 (55.00)	.05
High-density lipoprotein cholesterol (mg/dL)	46.57 (19.02)	45.19 (18.09)	.33

^a^CDHCS: customized digital health care service.

### Comparison of MSA and MS Types Before and After Intervention

The distribution of the risk group increased from 32 (50.8%) to 34 (54%) and that of the MS group decreased from 18 (28.6%) to 16 (25.4%). The mean MSA-CA before CDHCS was 2.20 (6.47) years, which decreased to 1.72 (5.80) years after CDHCS, showing a decrease of 0.48 years in the mean MSA-CA after the intervention. With respect to classification by MSA, the number of participants increased from 6 (9.5%) to 8 (12.7%) in the “good” MSA group and from 30 (47.6%) to 39 (61.9%) in the “average” MSA group, whereas the number of participants decreased from 27 (42.9%) to 16 (25.4%) in the “poor” MSA group (Table 2).

**Table 2:** Distribution and mean value related to metabolic syndrome variables.

Parameters		Before CDHCS^a^, n (%)	After CDHCS, n (%)	*P* value	
MSBA-CA^b^ (years), mean (SD)		2.20 (6.47)	1.72 (5.80)	.33	
**Classification by MSBA-CA**					.12
	Good	6 (9)	8 (13)		
	Average	30 (47)	39 (62)		
	Poor	27 (43)	16 (25)		
**Type of MS^c^**					.92
	Normal group	13 (21)	13 (20)		
	Risk group	32 (51)	34 (54)		
	MS group	18 (29)	16 (25)		

^a^CDHCS: customized digital health care service.

^b^MSBA-CA: (Metabolic syndrome biological age)–chronological age.

^c^MS: metabolic syndrome.

### Changes in Lifestyle Before and After Intervention

The mean frequency of exercise per week was 4.11 times before the intervention, which increased by 0.27 times to 4.38 times after the intervention. For smoking and drinking status, the number of nonsmokers increased from 58 (92.1%) to 59 (53.7%) and smokers decreased from 5 (7.9%) to 4 (6.3%). The number of nondrinkers decreased from 38 (60.3%) to 32 (50.8%) and drinkers increased from 25 (39.7%) to 31 (49.2%). The mean frequency of alcohol consumption per week decreased from 1.75 to 1.15 times. The number of participants who slept < 7 or > 8 hours decreased from 47 (74.6%) to 37 (58.7%), whereas the number of participants who slept for 7-8 hours increased from 16 (25.4%) to 26 (41.3%). The stress score changed from 3.04 to 3.03 points. The mean step count was 6425 steps in the first month of using CDHCS, which increased by 203 steps to 6628 steps in the third month. Finally, the number of participants with good dietary habits increased by 3 after the intervention (Table 3). While all lifestyle variables, except alcohol consumption, showed a tendency towards improvement, the differences were not statistically significant.

**Table 3:** Changes in lifestyle variables before and after CDHCS use.

Parameters		Before CDHCS^a^	After CDHCS	*P* value	
The number of exercises, mean (SD)		4.11 (2.29)	4.38 (1.87)	.11	
**Smoking state, n (%)**					.97
	Nonsmoking	58 (92.1)	59 (93)		
	Current smoker	5 (7.9)	4 (6)		
**Drinking state, n (%)**					.37
	Nondrinking	38 (60)	32 (50)		
	Current drinker	25 (39)	31 (49)		
Alcohol consumption per week (g/w), mean (SD)		0.67 (1.48)	0.44 (0.86)	.45	
**Sleeping time, n (%)**					.09
	Less than 7 or more than 8 hours	47 (74)	37 (58)		
	7-8 hours	16 (25)	26 (41)		
Stress score, mean (SD)		3.04 (0.91)	3.03 (0.92)	.88	
Step counts, mean (SD)		6425 (3262.32)	6628 (3141.00)	.02	
**Eating habit**					.85
	Good	35 (55)	38 (60)		
	Bad	28 (44)	25 (39)		

^a^CDHCS: customized digital health care service.

## Discussion

### Principal Findings

The objective of this pilot study was to verify the effects of using a CDHCS for 3 months on MS risk factors and lifestyle improvement among 63 participants. The results showed a tendency toward improvement in all parameters, except alcohol consumption, but the differences were not statistically significant. In the distribution of MS types, 2 out of 18 participants (11.1%) in the MS group were downgraded by one level to the risk group after the intervention. Of 27 participants, 9 (56.3%) in the “poor” group were upgraded to the “average” or “good” groups, which can be interpreted as a partial improvement in factors affecting MS.

According to previous studies that evaluated the effectiveness of digital health care on patients who are diabetic, patients who received 12 months of *untact* monitoring and health care coaching showed improvement in test parameters (FBS, hemoglobin A_1c_, glycated hemoglobin, total cholesterol, HDL-C, LDL-C, and TG). The results also confirmed significant improvement in clinical indicators in the group that received the service, compared to the control group that did not [19,22]. A previous study on hypertension reported improvement in BP levels after receiving digital health care intervention [21]. Similarly, another study on the effects of 12 weeks of digital health care use on the MS indicators and physical characteristics of MS reported statistically significant improvement in weight, BMI, WC, FBS, cholesterol, and TG, indicating that encouraging physical activity promotion through digital health care had a positive effect on clinical indicators related to MS [32]. Moreover, other MS studies concluded that the severity of MS was improved more effectively by digital health care use [33].

### Limitations and Strengths

Data for this study were collected in 2020 during the COVID-19 pandemic. At the time, the social distancing policy including restrictions on outdoor activities, visiting sports facilities, and private gatherings was being implemented. These restrictions may amount to a factor affecting the lifestyle and may have influenced the results of this study. Restrictions on outdoor activities naturally caused a decrease in the frequency of exercise and the number of steps. Our results showed a slight increase in the number of steps and frequency of exercise after using the CDHCS, but we expect that if there were no restrictions owing to COVID-19, the results would have changed. Meanwhile, prolongation of such a lifestyle pattern can act as a factor that causes significant levels of stress, depression, and helplessness among modern people facing tremendous changes in their daily lives, a phenomenon referred to as “*Corona Blue*” [34].

It was difficult to find statistical significance in this pilot study because of several factors. However, this study was significant in that it was the first to analyze the severity of MS and provide the overall health status, and aging to the users in an easy-to-understand manner. While many previous studies on digital health care applied health care coaching using SMS or telephone calls, this study used a mobile application that was developed based on big-data analysis research [31].

MS has many aspects that require improvements in daily life, and thus, long-term health care services rather than short-term medical services are required [35]. In this study, the normal group maintained its normal status without any change even after 3 months. It is important for the risk or MS group to improve and to help the normal group maintain its condition. Therefore, health care systems must focus on continuous care such as disease prevention and lifestyle improvement to increase the healthy life expectancy, while it is also important to provide adequate and effective health care services without visiting hospitals [28]. Effective digital health care can help individuals identify the risk factors for diseases to prevent them while also helping to prevent the onset of complications and symptom exacerbation [26-29].

The services help by providing continuous feedback, motivation, and encouragement for lifestyle and behavioral changes, and continuous interactions between health care coaching (apps) and users can lead to positive results by improving user compliance [22,36]. Activation of such interaction has some requirements; it needs to be cost-effective and customizable, and provide timely notifications.

Lastly, this study had some limitations. Relatively short-term effects were investigated. This was a pilot study; thus, an additional study will consider and supplement these points to demonstrate the effectiveness of a CDHCS with respect to significant improvement in MS and lifestyle. Moreover, the additional study will have a larger sample size and will extend the intervention period to increase the statistical power.

### Conclusion

The CDHCS used in this study identifies and analyzes lifestyle, exercise, habits, and current step count through a mobile phone, and based on this, provided customized real-time feedback and health guidance. These services served to encourage, remind, and motivate study subjects about health care. However, although no statistically significant variable was found in this study, it is thought that it may have been influenced by several external factors. Currently, the world is rapidly advancing to the era of the fourth industrial revolution owing to COVID-19, which renders the development of non–face-to-face medical and health care field as indispensable. Therefore, research in this field will make a great contribution to the development of digital health care by providing a foothold to deliver a high-quality personalized health care program with scientific evidence to healthy people and subjects with disease risk factors.

## Abbreviations

CDHCS: customized digital health care service
DBP: diastolic blood pressure
DTx: digital therapeutics
FBS: fasting blood sugar
HDL-C: high-density lipoprotein cholesterol
MS: metabolic syndrome
MSA-CA: metabolic syndrome age-chronological age
NHIS: National Health Insurance Service
SBP: systolic blood pressure
TG: triglyceride
WC: waist circumference

## Data Availability

The data sets generated during this study are not publicly available because consent was not obtained from participants to share the research data publicly; however, the data are available from the corresponding author upon reasonable request.
